# Supplementary material for: Precision-edited histone tails disrupt polycistronic gene expression controls in trypanosomes
Source: Nat Commun. 2025 Jul 4;16:6194. doi: 10.1038/s41467-025-61480-z (PMC12227686; doi:10.1038/s41467-025-61480-z)
Supplement: Supplementary file 1 — Supplementary Information [file 41467_2025_61480_MOESM1_ESM.pdf]

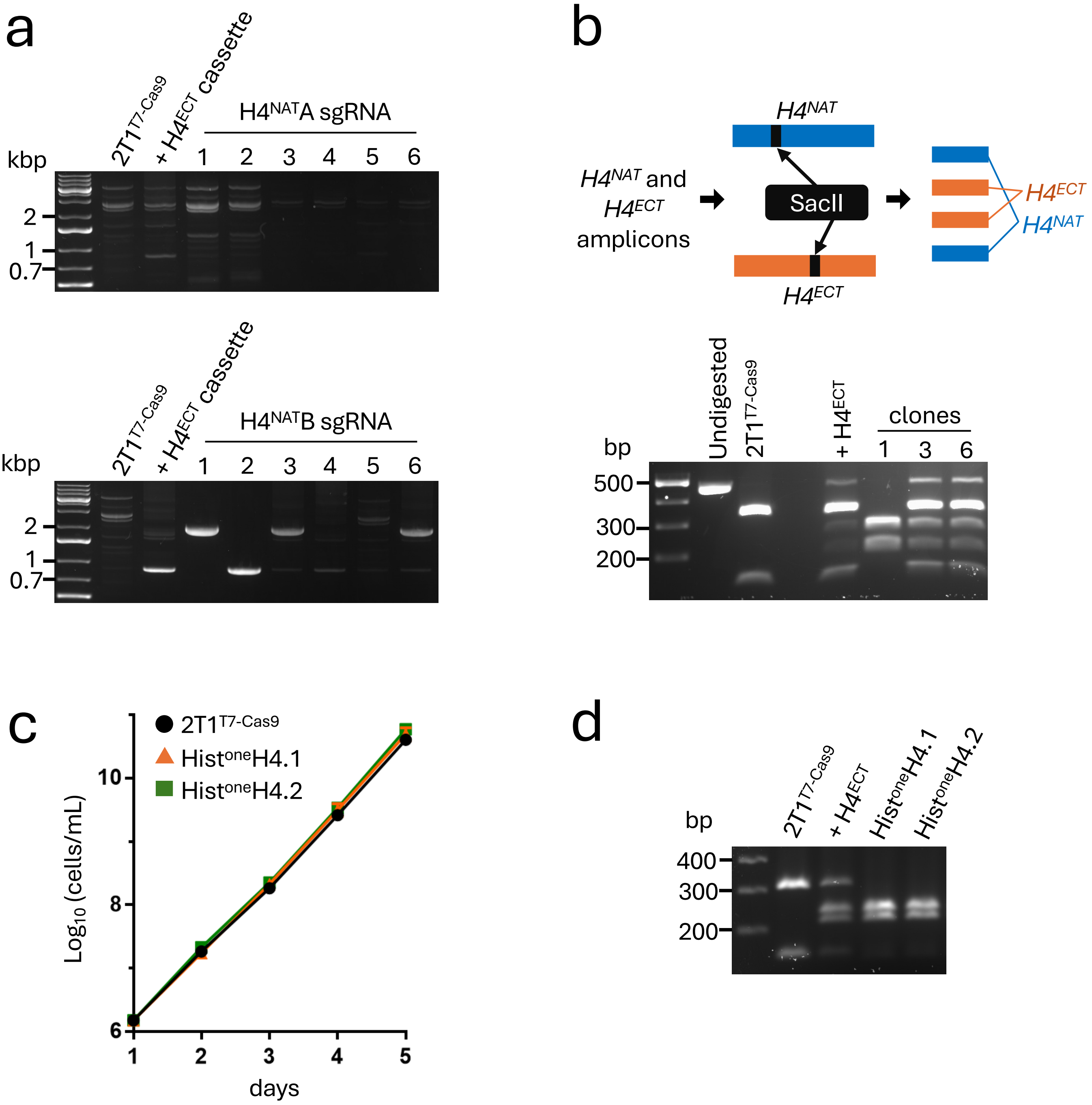

Supplementary Fig. 1: Generation and initial validation of hist^{one}H4 strains.

a PCR assay to test incorporation of the *NPT* cassette and *H4* array knockout in *T. brucei* strains expressing the *H4^{NAT}A* (upper panel) or *H4^{NAT}B* sgRNA (lower panel). Only the *H4^{NAT}B* sgRNA yielded correct incorporation, indicated by 2020 bp amplicon in clones 1, 3 and 6. **b** Native and ectopic *H4* genes were PCR-amplified from clones 1, 3 and 6, and digested with SacII. The gel shows that only clone 1 lacked native *H4* genes. The process was repeated to generate a second independent hist^{one}H4 strain. **c** The cumulative growth curves show that growth of the hist^{one}H4 strains is comparable to the parental 2T1$^{T7\text{-}Cas9}$ strain. Two technical replicates. Some error bars are obscured by the datapoints. **d** Native and ectopic *H4* transcripts were reverse-transcribed and PCR-amplified from both hist^{one}H4 strains and controls and digested with SacII. The gel indicates similar abundance transcripts from native and ectopic *H4* genes and only transcripts from the ectopic *H4* gene in the hist^{one}H4 strains, as expected.

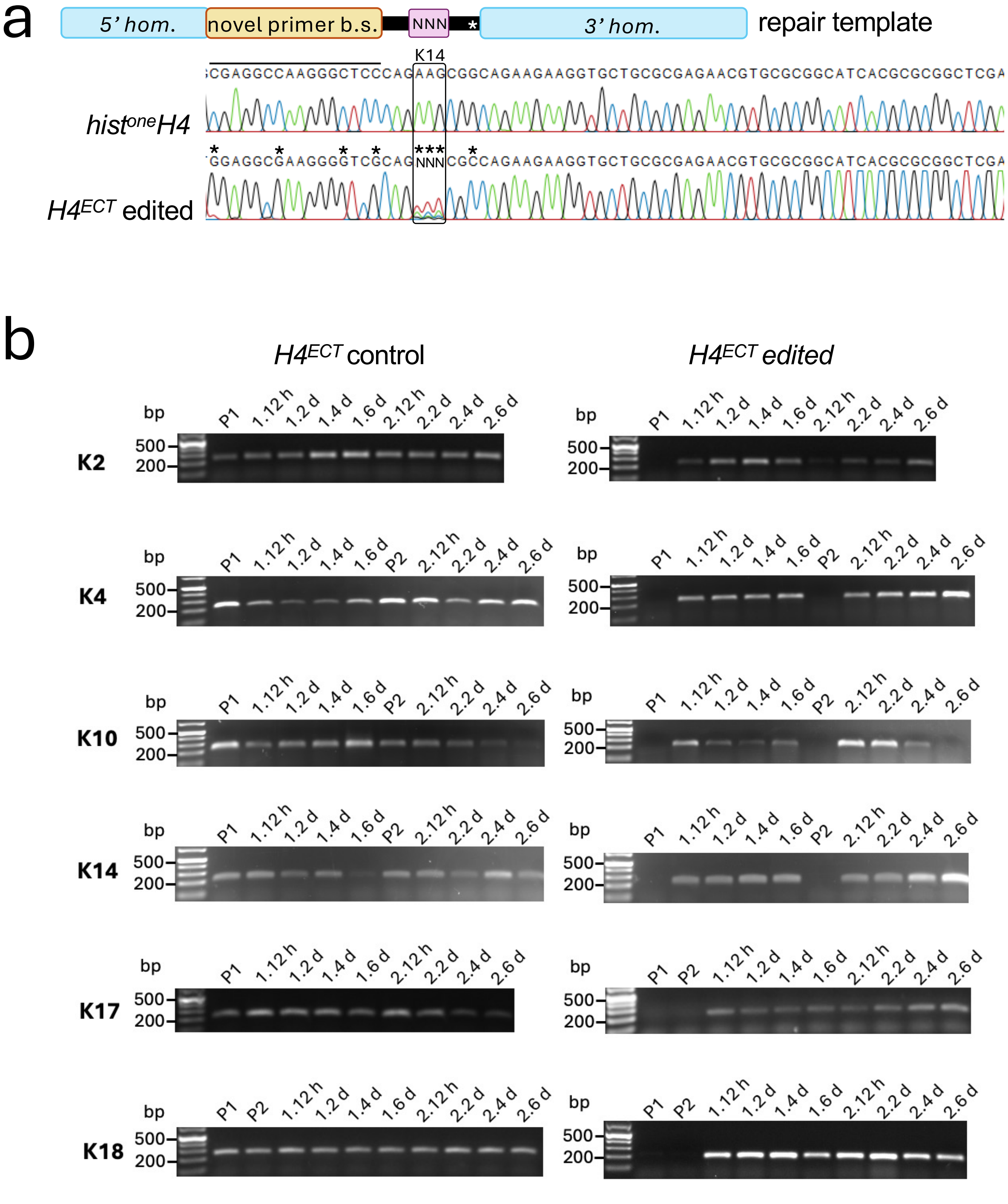

Supplementary Fig. 2: Ectopic H4 N-tail lysine mutagenesis.

a The sequence traces show a representative example of the unedited ectopic *H4* gene and an edited gene with a repair template illustrated above. Repair-templates contained 5' and 3' homology arms (5', 3' hom.), a series of synonymous edits designed to generate a novel primer binding site (b.s.), a degenerate codon (NNN, in this case at lysine 14), and a synonymous base edit distal to the primer b.s. (*). Asterisks above the edited sequence-trace highlight introduction of the desired base-edits. **b** The PCR assays show the presence of the ectopic *H4* gene in all samples across all six 6-day time-courses (left-hand panels) and, exploiting the novel primer binding-site, the presence of edited sequences following delivery of editing templates (right-hand panels). Data are shown for editing in both hist^{one}H4 strains expressing the H4^{ECT} targeting sgRNA, and in the unedited parent samples, P1 and P2. The amplicons shown on the right were deep-sequenced.

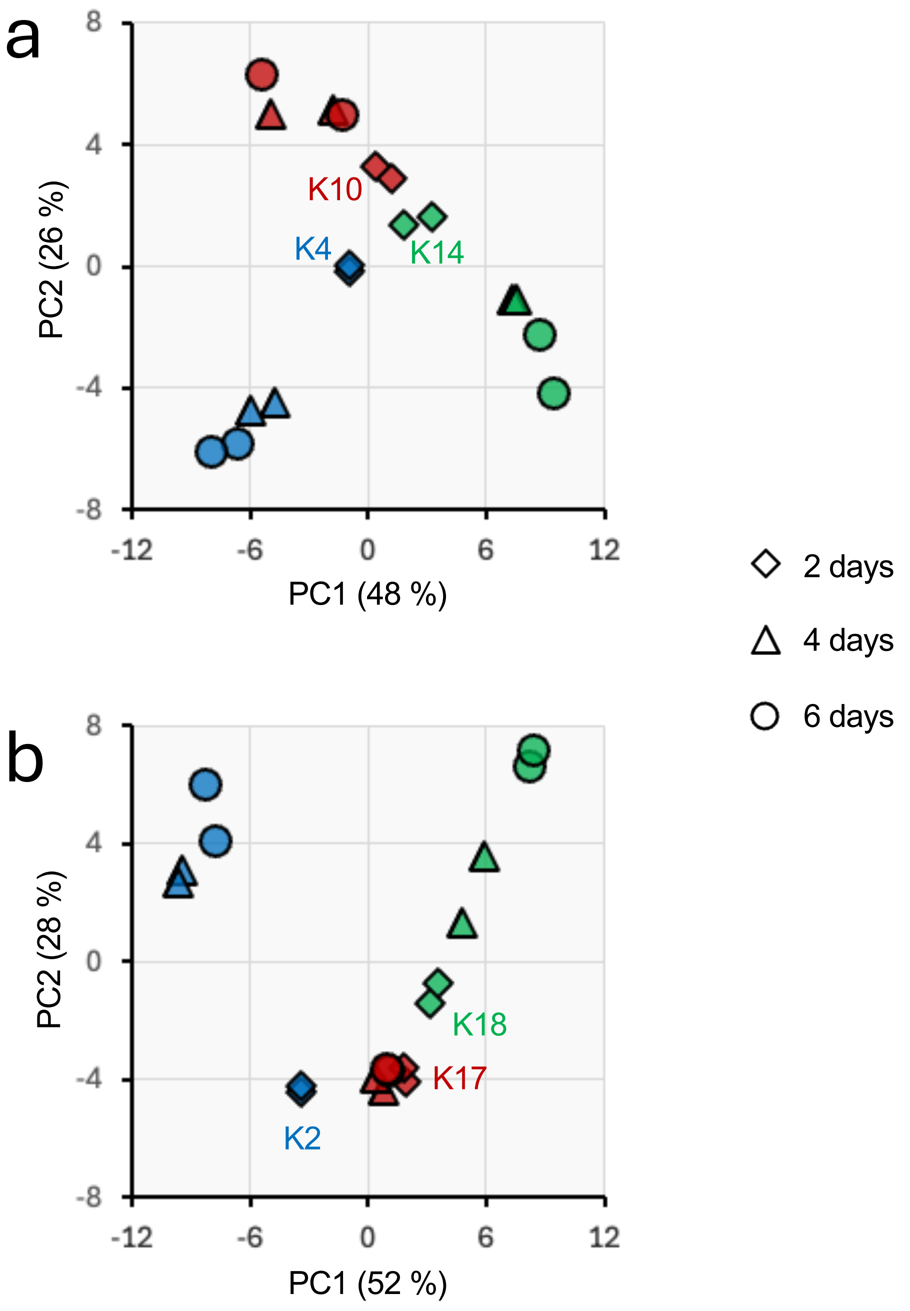

Supplementary Fig. 3: Replica edited libraries yielded highly consistent results.
a Relative read-counts for each codon (n = 64), and for each pair of replica edited libraries were assessed by principal component analysis at the timepoints indicated. Data are show for libraries edited at the $H4^{K4}$, $H4^{K10}$, or $H4^{K14}$ positions. **b** As in a above but for libraries edited at the $H4^{K2}$, $H4^{K17}$, and $H4^{K18}$ positions.

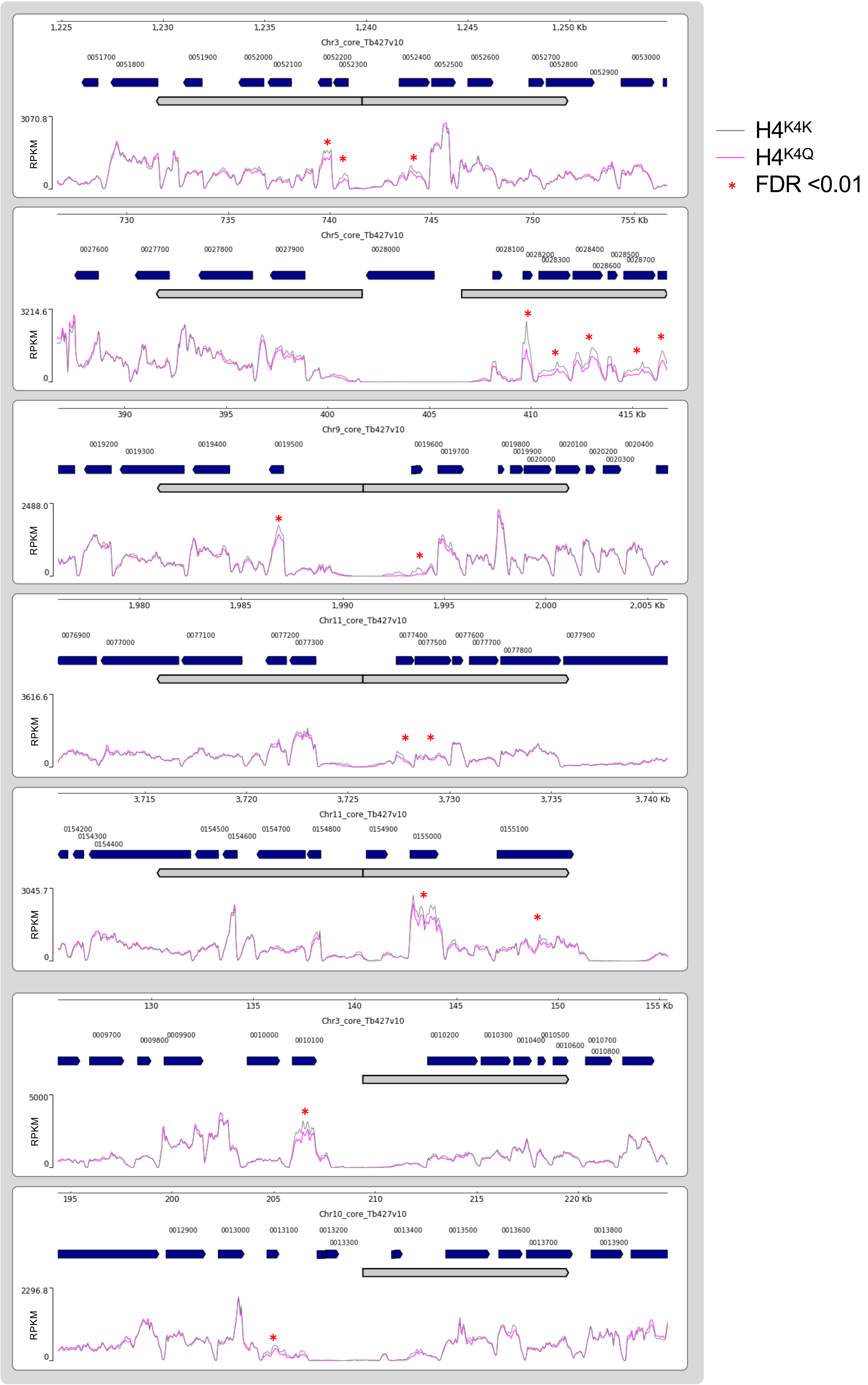

Supplementary Fig. 4: Expression of promoter-adjacent genes is disrupted in H4^{K4Q} mutants. Individual examples of promoter-proximal regions showing annotated CDSs in blue and 10 kbp promoter regions in grey; as defined by the upstream borders of H4^{K10} acetylation footprints. Five divergent start-sites and two non-divergent start-sites are shown. RPKM, Reads Per Kilobase per Million mapped reads.

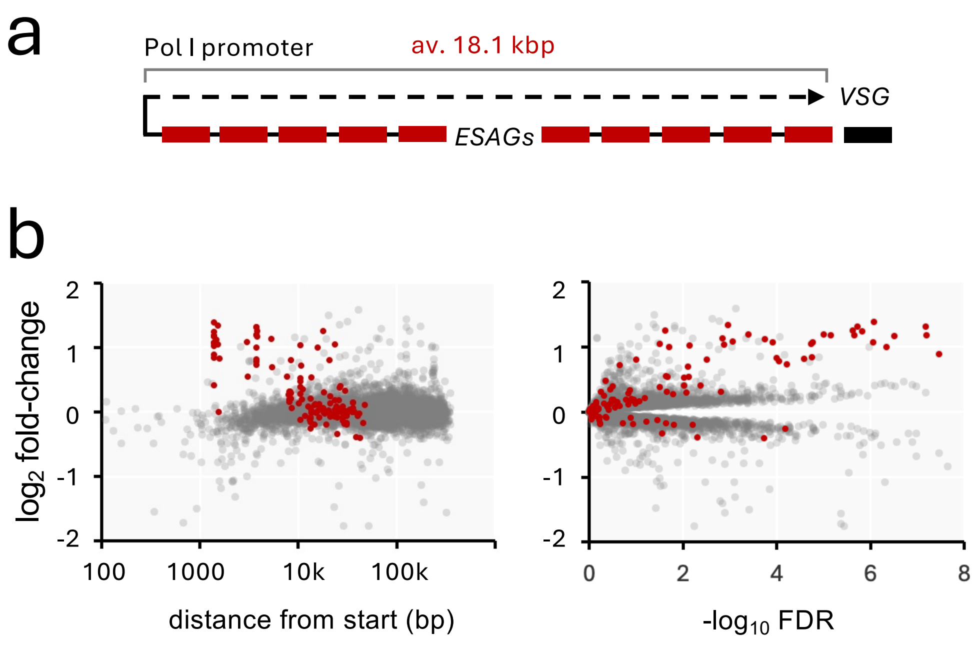

Supplementary Fig. 5: Expression of RNA polymerase I promoter-adjacent genes is disrupted in H4^{K4Q} mutants

a The schematic illustrates a canonical RNA polymerase I transcribed polycistronic VSG expression site in *T. brucei*. **b** RNA-seq analysis highlighting expression site associated genes. Distances from the promoter are shown on the x-axis (left-hand panel). FDR, False Discovery Rate.
