# Supplementary material for: Precision-edited histone tails disrupt polycistronic gene expression controls in trypanosomes
Source: Nat Commun. 2025 Jul 4;16:6194. doi: 10.1038/s41467-025-61480-z (PMC12227686; doi:10.1038/s41467-025-61480-z)
Supplement: Supplementary file 3 — Description of Additional Supplementary Files [file 41467_2025_61480_MOESM3_ESM.pdf]

Description of Additional Supplementary Files

File Name: Supplementary Data 1
Description: The “Supplementary Data 1” Excel file contains the following information:

- Oligonucleotides
- Editing templates
- Cell lines
- RNAseq +H4ECT vs 2T1T7-Cas9
- RNAseq histoneH4 vs +H4ECT
- RNAseq H4K4Q vs H4K4K
- Proteomics H4K4Q vs H4K4K
- Proteomics H4K14Q vs H4K14K

This information with more detail is also included in the Excel file itself.
