# Supplementary material for: Precision-edited histone tails disrupt polycistronic gene expression controls in trypanosomes
Source: Nat Commun. 2025 Jul 4;16:6194. doi: 10.1038/s41467-025-61480-z (PMC12227686; doi:10.1038/s41467-025-61480-z)
Supplement: Supplementary file 5 — Reporting Summary [file 41467_2025_61480_MOESM5_ESM.pdf]

nature portfolio

Corresponding author(s): David Horn

Last updated by author(s): Jun 13, 2025

Reporting Summary

Nature Portfolio wishes to improve the reproducibility of the work that we publish. This form provides structure for consistency and transparency in reporting. For further information on Nature Portfolio policies, see our Editorial Policies and the Editorial Policy Checklist.

Statistics

For all statistical analyses, confirm that the following items are present in the figure legend, table legend, main text, or Methods section.

n/a	Confirmed	
☐	☒	The exact sample size (n) for each experimental group/condition, given as a discrete number and unit of measurement
☐	☒	A statement on whether measurements were taken from distinct samples or whether the same sample was measured repeatedly
☐	☒	The statistical test(s) used AND whether they are one- or two-sided<br>*Only common tests should be described solely by name; describe more complex techniques in the Methods section.*
☒	☐	A description of all covariates tested
☒	☐	A description of any assumptions or corrections, such as tests of normality and adjustment for multiple comparisons
☐	☒	A full description of the statistical parameters including central tendency (e.g. means) or other basic estimates (e.g. regression coefficient) AND variation (e.g. standard deviation) or associated estimates of uncertainty (e.g. confidence intervals)
☐	☒	For null hypothesis testing, the test statistic (e.g. F, t, r) with confidence intervals, effect sizes, degrees of freedom and P value noted<br>*Give P values as exact values whenever suitable.*
☒	☐	For Bayesian analysis, information on the choice of priors and Markov chain Monte Carlo settings
☒	☐	For hierarchical and complex designs, identification of the appropriate level for tests and full reporting of outcomes
☒	☐	Estimates of effect sizes (e.g. Cohen's d, Pearson's r), indicating how they were calculated

Our web collection on statistics for biologists contains articles on many of the points above.

Software and code

Policy information about availability of computer code

Data collection	No previously unreported custom computer code or algorithm was central to data collection reported in the paper.
Data analysis	OligoSeeker Python package designed to process paired FASTQ files and count occurrences of specific codons. https://github.com/mtinti/OligoSeeker / Tinti, M. OligoSeeker. https://doi.org/10.5281/zenodo.15011916 (2025).

For manuscripts utilizing custom algorithms or software that are central to the research but not yet described in published literature, software must be made available to editors and reviewers. We strongly encourage code deposition in a community repository (e.g. GitHub). See the Nature Portfolio guidelines for submitting code & software for further information.

Data

Policy information about availability of data

All manuscripts must include a data availability statement. This statement should provide the following information, where applicable:

- Accession codes, unique identifiers, or web links for publicly available datasets
- A description of any restrictions on data availability
- For clinical datasets or third party data, please ensure that the statement adheres to our policy

Data availability: The genomic, transcriptomic, and amplicon-sequencing data have been deposited in the Sequence Read Archive under BioProject PRJNA1234166 [https://www.ncbi.nlm.nih.gov/sra/PRJNA1234166]. The mass spectrometry proteomics data have been deposited to the ProteomeXchange Consortium via the PRIDE partner repository under accession code PXD061709 [https://www.ebi.ac.uk/pride/archive?keyword=PXD061709]. Source data are provided with this paper.

Research involving human participants, their data, or biological material

Policy information about studies with human participants or human data. See also policy information about sex, gender (identity/presentation), and sexual orientation and race, ethnicity and racism.

Reporting on sex and gender	This study did not involve any human participants. This information is not relevant and has not been collected.
Reporting on race, ethnicity, or other socially relevant groupings	This study did not involve any human participants.
Population characteristics	This study did not involve any human participants.
Recruitment	This study did not involve any human participants.
Ethics oversight	This study did not involve any human participants.

Note that full information on the approval of the study protocol must also be provided in the manuscript.

Field-specific reporting

Please select the one below that is the best fit for your research. If you are not sure, read the appropriate sections before making your selection.

- [x] Life sciences
- [ ] Behavioural & social sciences
- [ ] Ecological, evolutionary & environmental sciences

For a reference copy of the document with all sections, see nature.com/documents/nr-reporting-summary-flat.pdf

Life sciences study design

All studies must disclose on these points even when the disclosure is negative.

Sample size	Two biological replicates were used for saturation mutagenesis and multiplex fitness-profiling. Three technical replicates were always used for proteomic analysis and RNA sequencing; three biological replicates of the H4K4Q strain were used.
Data exclusions	No data were excluded from the analyses.
Replication	We performed several independent experiments in some cases (PCRs, restriction digests, protein blotting, Southern blotting, saturation mutagenesis). We did not identify any non-replicable results.
Randomization	No randomization applied.
Blinding	No blinding applied.

Reporting for specific materials, systems and methods

We require information from authors about some types of materials, experimental systems and methods used in many studies. Here, indicate whether each material, system or method listed is relevant to your study. If you are not sure if a list item applies to your research, read the appropriate section before selecting a response.

Materials & experimental systems

n/a	Involved in the study	
☐	☒	Antibodies
☐	☒	Eukaryotic cell lines
☒	☐	Palaeontology and archaeology
☒	☐	Animals and other organisms
☒	☐	Clinical data
☒	☐	Dual use research of concern
☒	☐	Plants

Methods

n/a	Involved in the study	
☒	☐	ChIP-seq
☒	☐	Flow cytometry
☒	☐	MRI-based neuroimaging

Antibodies

Antibodies used	Primary antibodies:<br>rabbit anti-H4K4 acetylation, George Cross laboratory (Rockefeller University), Siegel et al., 2008.<br>rabbit anti-non-acetylated H4K4, George Cross laboratory (Rockefeller University), Siegel et al., 2008.<br>rabbit anti-H4K10 acetylation, George Cross laboratory (Rockefeller University), Siegel et al., 2008.

	mouse anti-EF1α, Sigma-Aldrich, cat. num.: 05-235, RRID:AB_309663<br>Secondary antibodies:<br>goat α-rabbit IRDye800, Li-COR, cat. num.: 925-32211, RRID:AB_2651127<br>goat α-mouseIRDye680, Li-COR, cat. num.: 925-68070, RRID:AB_2651128
Validation	Antibodies detecting H4K4 acetylation, non-acetylated H4K4, and H4K10 acetylation were generated and validated by George Cross's laboratory (Rockefeller University) and published in Siegel et al., 2008.

Eukaryotic cell lines

Policy information about cell lines and Sex and Gender in Research

Cell line source(s)	Trypanosoma brucei brucei Lister 427 was originally obtained from Prof. George Cross (Rockefeller University, NYC, USA). The 2T1.T7-Cas9 strain was generated in Prof. David Horn's lab and previously published (Rico et al., 2018). Other cell lines were based on 2T1.T7-Cas9 and generated by the authors.
Authentication	HistONE c1 and c2 cell lines were authenticated by Southern blotting, whole genome sequencing, RNA sequencing and proteomic analysis. Additional genetic editing in intermediate cells lines, and H4K4 and H4K14 mutants were confirmed by Sanger sequencing.
Mycoplasma contamination	Mycoplasma contamination check carried our approx. every 5 years - no positive results from those tests to date.
Commonly misidentified lines<br>(See ICLAC register)	None of the used cell lines are commonly misidentified.

Plants

Seed stocks	Plants were not used in this study.
Novel plant genotypes	Plants were not used in this study.
Authentication	Plants were not used in this study.
